# Age-Associated Metabolic and Morphologic Changes in Mitochondria of Individual Mouse and Hamster Oocytes

**DOI:** 10.1371/journal.pone.0064955

**Published:** 2013-05-31

**Authors:** Fatma Simsek-Duran, Fang Li, Wentia Ford, R. James Swanson, Howard W. Jones, Frank J. Castora

**Affiliations:** 1 Department of Physiological Sciences, Eastern Virginia Medical School, Norfolk, Virginia, United States of America; 2 Department of Biological Sciences, Old Dominion University, Norfolk, Virginia, United States of America; 3 Jones Institute for Reproductive Medicine, Department of Obstetrics and Gynecology, Eastern Virginia Medical School, Norfolk, Virginia, United States of America; Institute of Zoology, Chinese Academy of Sciences, China

## Abstract

**Background:**

In human oocytes, as in other mammalian ova, there is a significant variation in the pregnancy potential, with approximately 20% of oocyte-sperm meetings resulting in pregnancies. This frequency of successful fertilization decreases as the oocytes age. This low proportion of fruitful couplings appears to be influenced by changes in mitochondrial structure and function. In this study, we have examined mitochondrial biogenesis in both hamster (*Mesocricetus auratus* ***)*** and mouse (*Mus musculus*) ova as models for understanding the effects of aging on mitochondrial structure and energy production within the mammalian oocyte.

**Methodology/Principal Findings:**

Individual metaphase II oocytes from a total of 25 young and old mice and hamsters were collected from ovarian follicles after hormone stimulation and prepared for biochemical or structural analysis. Adenosine triphosphate levels and mitochondrial DNA number were determined within individual oocytes from young and old animals. In aged hamsters, oocyte adenosine triphosphate levels and mitochondrial DNA molecules were reduced 35.4% and 51.8%, respectively. Reductions of 38.4% and 44% in adenosine triphosphate and mitochondrial genomes, respectively, were also seen in aged mouse oocytes. Transmission electron microscopic (TEM) analysis showed that aged rodent oocytes had significant alterations in mitochondrial and cytoplasmic lamellae structure.

**Conclusions/Significance:**

In both mice and hamsters, decreased adenosine triphosphate in aged oocytes is correlated with a similar decrease in mtDNA molecules and number of mitochondria. Mitochondria in mice and hamsters undergo significant morphological change with aging including mitochondrial vacuolization, cristae alterations, and changes in cytoplasmic lamellae.

## Introduction

A growing body of evidence suggests that age-related sub-fertility in mammals is primarily linked to oocyte senescence and the resultant changes in oocyte structure and function that accumulate over time. Recent studies have shown that low quality oocytes have age-related dysfunctions including reduced mitochondrial membrane potential, increased mitochondrial DNA (mtDNA) damage and chromosomal aneuploidies, a higher incidence of apoptosis, and changes in mitochondrial gene expression [1], [2]. An inability of mitochondria to amplify and/or to accumulate adenosine triphosphate (ATP) has been linked to developmental abnormality or arrest. [3] Any decline in the amount of ATP production would potentially have dramatic effects on the specific functions of the mitochondria such as membrane transport, nutrient synthesis, and mechanical work. In addition, the question of the numerical size of a normal mitochondrial complement in the mature oocyte is considered a critical factor in the determination of oocyte and embryo developmental competence [4], [5]. Thus, ATP levels and the number of mtDNA molecules are critical parameters affecting the overall pregnancy potential of individual mammalian oocytes.

The mouse and hamster have been especially useful models for elucidating various aspects of oogenesis and reproductive development including, in hamster, the localization and activity of mitochondria during maturation and fertilization [6], the role of estradiol-17 beta in the development of primordial follicles [7], the tridimensional structure of the zona pellucida [8] and a variety of other fundamental aspects of reproductive physiology, oncology, genetics and virology [9]. Likewise, the mouse model has been used to reveal mechanisms regulating major reproductive and developmental events [10], global oocyte gene expression [11], RNA silencing pathways [12], intra-oocyte redox state [13], and critical events during meiotic initiation in the ovary, follicle formation, and oocyte growth and maturation [14].

In this paper, we expand on our preliminary observations previously published in abstract form [15], [16]. Although there are an increasing number of reports of changes in ATP and mtDNA levels in other animals [17]–[21], our work is novel because it is the first to measure both ATP and mtDNA levels in the same individual oocyte. With this approach, we were able to demonstrate a small but significant correlation between the number of mitochondrial genomes and the level of ATP in these individual oocytes from both mouse and hamster. Furthermore, we also show that a significant reduction in oocyte ATP level and mtDNA number occurs during mouse and hamster aging. These age-related deficits are also reflected in ultrastructural changes in the oocyte, particularly in the cytoplasmic lamellae and mitochondrial cristae.

## Results

### ATP Levels in Individual Mouse and Hamster Oocytes

As one measure of mitochondrial function, we determined the levels of intracellular ATP within individual mouse oocytes obtained from both young (2–4 month old) and old (10–12 months old) animals using a highly sensitive chemiluminescent assay.

**Figure 1:**
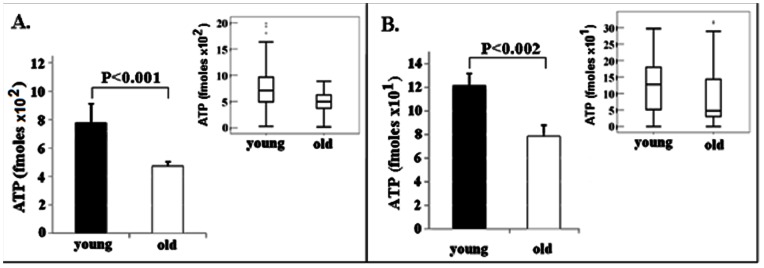
Measurement of ATP levels (fmoles) in individual mouse (A) and hamster (B) oocytes. Note that there is a 38.4% decrease in ATP levels in old mice vs. young mice (*P*<0.0001) and a 35.4% drop in ATP in old vs. young hamster oocytes. (*P* = 0.002). Data are also presented as median with 10^th^, 25^th^, 75^th^, and 90^th^ percentiles and outliers in whisker plot inserts. Statistical analysis was performed by Student T-test and a *P* value less than 0.05 was considered significantly different between the groups.

Fig. 1A shows the results of these assays. ATP levels in 117 individual mouse eggs (56 from 5 young animals (2–4 months of age) and 61 from 6 old mice (10–12 months of age) were measured. We found that the young mice possess on average 767+/−65 fmoles ATP per oocyte while the old mice contain 472+/−28 fmole ATP (p<0.0001). Thus, individual mouse oocytes displayed, on average, a 38.4% decrease in ATP levels with aging.

Fig. 1B shows the results of ATP assays from young and old hamster oocytes. We have measured ATP levels in 163 individual hamster oocytes (90 from 6 young animals and 73 from 7 old animals). We found that the young hamster oocytes contain an average of 121.5+/−10 fmoles ATP (p = 0.002) while the old hamster oocytes possess an average of 78.45+/−9.5 fmoles. Similar to the observations with individual mouse oocytes, there is a decrease of 35.4% in the average level of ATP in individual old hamster oocytes compared to that in oocytes from young animals.

### Total mtDNA Number in Individual Mouse and Hamster Oocytes

It is estimated that there are 1–2 mtDNA molecules per mitochondrion in the mammalian oocyte [5]. As oocyte quality has been linked to the size of the mitochondrial cohort [22], we performed quantitative real time PCR using recombinant plasmid DNA to construct the standard curve in order to determine the number of mtDNA genomes in the same mouse and hamster oocytes used for the ATP determinations. We felt that the circular nature of the recombinant substrate would more accurately reflect the topology of the endogenous mtDNA and thus give a true measure of mtDNA number.

**Figure 2:**
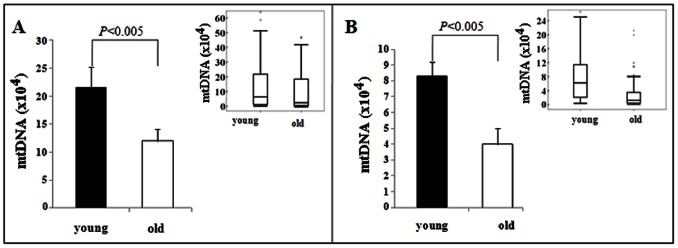
Real-time PCR analysis of mtDNA copy numbers in individual mouse (A) and hamster (B) oocytes. mtDNA copy numbers were significantly lower in old oocytes compared to young oocytes in both mice and hamsters. There is a 44% decrease in mtDNA number in aged mouse oocytes and a 51.8% decrease in aged hamster oocytes. Data were also presented as median with 10^th^, 25^th^, 75^th^, and 90^th^ percentiles and outliers shown in whisker plot inserts.

Fig. 2A shows the results of the determination of mtDNA number and, indirectly, mitochondria in individual mouse oocytes. We retrieved 154 mouse oocytes (80 from 5 young animals and 74 from 6 old animals). We found that the ova from young mice possess an average of 214,680+/−39,963 mtDNA copies per oocyte while the old mice have oocytes that contain an average of 120,135+/−21,461 mtDNA copies. Thus, there is a decrease of 44% (p = 0.039) in the average number of mtDNA molecules in individual old mouse oocytes compared to that in individual ova of young mice.

Fig. 2B shows a similar analysis of mtDNA number in individual hamster oocytes from young (4 mo) and old (12–14 mo) animals. MtDNA copy number in 118 hamster oocytes (63 from 7 young animals and 55 from 7 old animals) were measured. We found that the young hamsters contain an average of 83,134+/−10,114 mtDNA copies per oocyte while the individual old hamster oocytes possess an average of 40,099+/−8,622 mtDNA copies. This reflects a decrease of 51.8% (p = 0.0015) in the average number of mtDNA genomes in individual old hamster oocytes compared to young hamster oocytes.

### Correlation of ATP Levels and mtDNA Number in Individual Mouse and Hamster Oocytes

**Figure 3:**
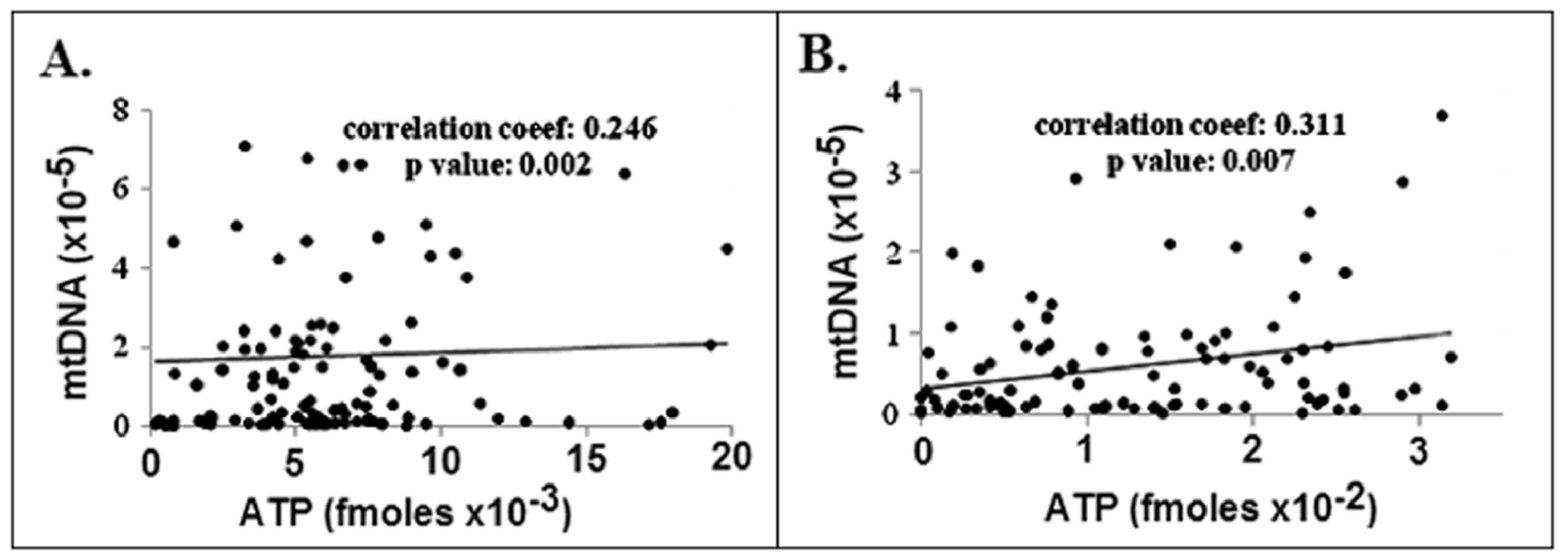
Correlation of ATP level and mtDNA copy numbers within single oocytes. Left panel (A), mouse oocytes and right panel (B), hamster oocytes. Statistical analysis was done by the Pearson correlation test.

Our results indicate significant reductions in both the ATP levels and mtDNA numbers in both old mouse and hamster oocytes relative to those quantities in young animals when the average values are compared. In addition, Fig. 3 shows that there is a small (r = 0.246) to medium (r = 0.311, p = 0.03) correlation of ATP level and mtDNA number within individual mouse and hamster oocytes, respectively, that is statistically significant. These correlations suggest that a low number of mitochondrial genomes, and, hence, mitochondria, reflects a low level of ATP in an oocyte, whether that oocyte is from a young or old animal.

### Morphological Analysis of Young and Old Mouse and Hamster Oocytes by TEM

**Figure 4:**
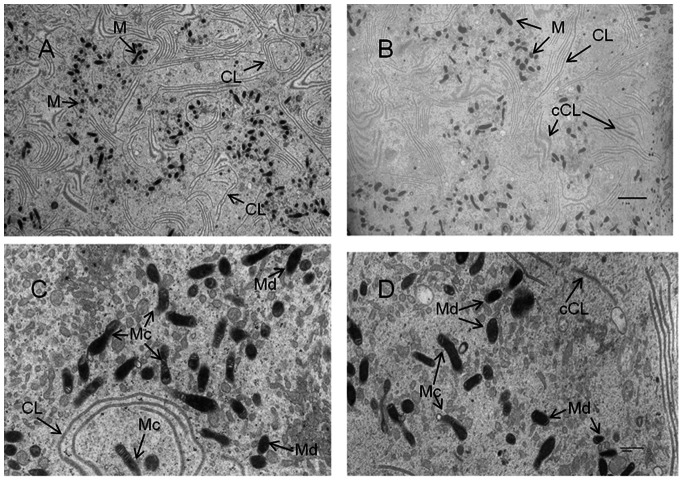
Ultrastructure of hamster MII oocytes. A and B are representative TEM micrographs at 2,000X magnification, showing various cytoplasmic lamellae (CL) and numerous mitochondria (M) in this oocyte from a young animal (A) compared to the number in an oocyte from an old hamster (B). C and D are representative TEM micrographs of oocytes from young (C) and old (D) hamsters at 10,000X magnification. Note the dark mitochondria (Md) and mitochondria with clear cristae (Mc). The old ova also showed the presence of collapsed cytoplasmic lamellae (cCL). (Bar: A&B, 2 µm; C&D, 500 nm).

The TEM analysis of mouse and hamster oocytes yielded interesting but mixed results. Fig. 4 shows representative electron micrographs obtained from analysis of individual hamster oocytes. From 10 oocytes retrieved from 5 young hamsters (Fig. 4A), we counted 683 total mitochondria from 10 vision fields (average number = 68.3±2.3) using ImageJ software to quantitate according to a method described by Weibel et al. [23]. From 10 oocytes collected from 5 old hamsters (Fig. 4B), we counted a total of 593 mitochondria within 10 vision fields (average number = 59.3±4.5), a statistically significant decrease of 15% (t-test, p<0.05) in mitochondrial number in aged vs young individual hamster oocytes. These TEM results support the observed age-related decrease in mitochondrial number detected by real-time PCR above.

Other differences in mitochondrial morphology were also observed by TEM. For example, many mitochondria could be seen to have electron dense matrices (Md) while other mitochondria had a much less electron dense matrix with more clearly visible cristae (Mc, containing more than 3 visible cristae). Oocytes from young hamsters (Fig. 4C) displayed a significantly higher percentage of Mc (54.1% ±2.7; 80 Mc and 68 Md mitochondria) relative to the percentage of Mc in ova from old hamsters (38.9% ±1.5; 75 Mc and 118 Md mitochondria) (p<0.001)(Fig. 4D). A similar observation has been reported in human oocytes [24], [25]. In addition, the hamster oocyte has clear regions of a conformational form of smooth endoplasmic reticulum described as cytoplasmic lamellae (CL) by Weakley [26]. There appear to be many regions of collapsed cytoplasmic lamellae (cCL) visible in the old hamster oocytes whereas there is no evidence of cCL in the young oocytes.

**Figure 5:**
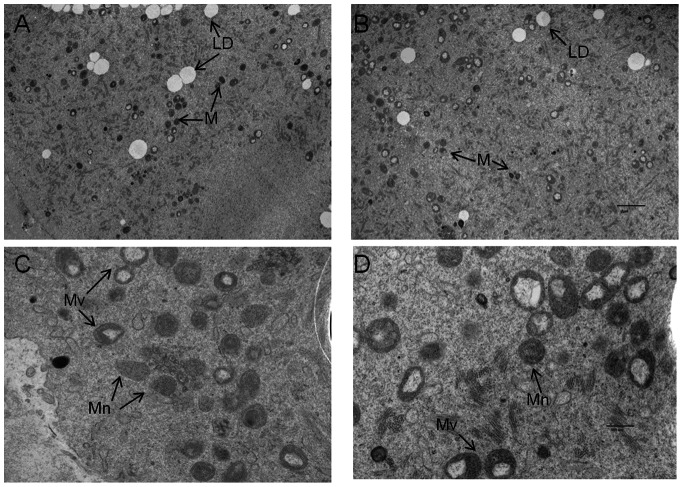
Ultrastructure of mouse MII oocytes. A and B are representative TEM micrographs at 3,000X magnification. Note the mitochondria (M) and lipid droplets (LD). C and D are representative TEM micrographs from young (C) and old (D) mice at 12,000X magnification. Note the normal (Mn) and vacuolated (Mv).mitochondria (Bar: A&B, 2 µm; C&D, 500 nm).

Fig. 5 shows representative TEM analysis of individual young and old mouse ova. We found, from 11 vision fields from 11 young mouse oocytes, a total of 317 mitochondria or an average of 28.8±3.5 mitochondria per individual oocyte (Fig. 5A). From a similar number of vision fields from 11 old mouse oocytes (Fig. 5B) we found a total of 252 mitochondria (average number = 22.9±3.1). However, this 25.8% decrease in the number of mitochondria in the aged mouse oocyte is not statistically significant as the p-value using a two-tailed, two-sample unequal variance t-test is 0.11.

Interestingly, upon higher magnification of these young (Fig. 5C) and old (Fig. 5D) mouse oocytes, it can be seen that the mitochondria appear as two morphologic types: (1) normal electron density with defined cristae (Mn) and (2) vacuolated (Mv). The percentage of vacuolated mitochondria is significantly higher in old mouse oocytes (50.9% ±3.1 per vision field; 128 Mv and 124 Mn mitochondria) compared to that in young mouse ova (34.1% ±1.6 per field; 108 Mv and 209 Mn mitochondria) (p<0.0005).

## Discussion

We have shown that both ATP content and mtDNA number decrease as the mouse and hamster oocyte ages, which agrees with results seen by others in a number of mammalian species, including human, rat, pig, cow and mouse [17]–[21] and with our previously published preliminary reports [15], [16]. In these published studies from other laboratories, ATP and mtDNA numbers have either been estimated as averages of groups of oocytes or individual oocytes have been used either for ATP measurements or mtDNA determinations but not for both simultaneously. In addition to demonstrating these age-related changes in ATP and mtDNA for the first time in the hamster, another novel feature of this work is the linkage between ATP levels and mtDNA numbers. By studying sufficient numbers of individual mouse and hamster oocytes, we have been able to demonstrate a statistically significant correlation within the same single oocyte between mtDNA numbers and the levels of ATP, i.e., as the number of mitochondrial genomes decreases with age, the intracellular ATP level within that oocyte also decreases. Furthermore, these biochemical changes in genome number and ATP level coincide with observed structural changes in the aged mouse and hamster oocytes. Iwata et al [20] looking at maternal age and changes in mtDNA, ATP and IVF outcomes in bovine oocytes concluded that all these parameters were affected by maternal age but that there was no clear relationship between ATP content and mtDNA number. The positive correlation that we see between mtDNA numbers and ATP levels could be the result of differences in the reproductive physiology of the different animals utilized in these two studies or could be a consequence of differences in the experimental protocols. Iwata et al pooled 10 bovine oocytes in their samples to be analyzed whereas we analyzed mtDNA and ATP within the same individual oocytes.

Mitochondrial activity is responsible for ATP production and energy accumulation during oogenesis, which is a crucial factor for successful development [3], [27]. Adequate ATP reserves in oocytes and embryos are critical for normal nucleic acid and protein synthesis, and energy levels have been suggested to be an indicator of the developmental potential of mouse [28], [29] and bovine embryos [30]. Furthermore, variations in ATP concentrations appear to be associated with embryo developmental competence in humans [31]. Recently, blastocyst formation has been linked to the number of mitochondria in the oocyte [32], where the average mtDNA copy number was significantly lower in cohorts of oocytes suffering from fertilization failure than cohorts with a normal fertilization. The quantity of mitochondria in the oocyte affects its ability to produce ATP [28], to escape atresia [17], and to support embryo development [33].

We also observed alterations in the morphology of mitochondria and the cytoplasmic lamellae in aged oocytes relative to young oocytes as evidenced by TEM analysis. Peluso and Butcher [34] reported that mitochondrial ultrastructure changed from a spherical to an elongated shape during *in vivo* ageing of rat oocytes. Similar changes to mitochondrial shape were also observed during *in vitro* ageing of porcine oocytes [35]. These earlier observations are concordant with our findings of spherical mitochondria in the young hamster oocytes with a greater proportion of elongated mitochondria evident in the aged hamster oocytes.

Relocation of mitochondria may be indicative of alterations in ATP production during oocyte ageing. Reorganization of mitochondria correlates with increased ATP content in bovine oocytes during *in vivo* maturation [36]. In addition, lipid droplets, considered to be a source of energy, have been seen to undergo significant changes in ultrastructure during porcine oocyte ageing. We did not notice any significant changes in the number or morphology of lipid droplets when comparing young and old hamster oocytes.

In all mammals, the mitochondria of metaphase II oocytes are small spherical organelles with a few cristae surrounding the electron-dense matrix. This aspect suggests a relatively weak energetic activity and low ATP production [37]. A reduction in oocyte/embryo ATP contents with age may inhibit protein synthesis and cellular functions including mitosis, compaction, blastocoel formation and hatching, and so delay and/or arrest embryo development. In fact, in the mouse there is a positive correlation between the amount of ATP in oocytes/embryos and the proportion of those oocytes/embryos reaching the blastocyst stage [27] as well as the proportion reaching the expanded and hatched blastocyst stage [38]. We see a similar relationship in our hamster model (unpublished observation). Furthermore, in humans an association between ATP content of oocytes and later embryo potential for implantation and/or development *in vivo* has been reported [39], [40]. Future studies with the hamster or mouse model could be directed toward elucidating these relationships between mitochondrial number and energy content and the processes of fertilization, implantation and development. It should be noted that recently it has been reported that ovarian hormonal hyperstimulation can adversely affect oocyte mitochondrial function, i.e., inhibiting mtDNA replication and decreasing inner membrane potential, compared to naturally maturation of oocytes [41]. Although this observation demands further investigation which may impact on future IVF procedures, we believe the similar hyperstimulation treatments provided to both our young and old mice and hamsters removes any bias anticipated from such treatment.

## Materials and Methods

### Animals and Reagents

#### Ethics statement

This study was carried out in strict accordance with the recommendations in the Guide for the Care and Use of Laboratory Animals of the National Institutes of Health. The protocols described were approved by the Institutional Animal Care and Use Committees (IACUC) at Eastern Virginia Medical School and Old Dominion University (Permit Numbers: TI-004 and 07-016). All surgery was performed under sodium pentobarbital anesthesia, euthanasia was performed by sodium pentobarbital overdose followed by pneumothorax and all efforts were made to minimize suffering.

Female B6CBAF1/J mice were purchased from Jackson Laboratory (Bar Harbor, ME). Young mice were purchased at 2 months of age while old mice were 10–16 months old when purchased. If not used immediately, the mice were typically maintained on site for 1–2 months before being terminated. The mouse media was modified Krebs Bicarbonate Buffered medium (mKBB), consisting of 94.01 mM NaCl, 4.78 mM KCl, 1.71 mM CaCl_2_, 1.18 mM KH_2_PO_4_, 1.19 mM MgSO_4_, 25.1 mM NaHCO_3_, 0.5 mM sodium pyruvate, 21.36 mM sodium lactate, 5.56 mM glucose, 4 mg/mL albumin, 50 IU/mL penicillin, 50 IU/mL streptomycin.

Female golden Syrian hamsters were purchased from Harlan Laboratories (Indianapolis, IN). Young hamsters were purchased at 3–4 months of age and terminated by age 4–8 months while old hamsters were purchased at 11–12 months of age and terminated by 12–16 months of age. The hamster media was modified Biggers, Whitten, Whittingham (BWW) Medium [42] consisting of 94.59 mM NaCl, 4.78 mM KCl, 24 µM CaCl_2_, 1.19 mM KH_2_PO_4_, 1.19 mM MgSO_4_, 25.07 mM NaHCO_3_, 0.25 mM sodium pyruvate, 23.02 mM sodium lactate, 5.56 mM glucose, 3 mg/mL albumin, 100 IU/mL penicillin, 100 IU/mL streptomycin.

Because of a limited or absent response to the hormonal hyperstimulation by the older mice and hamsters, the oocytes used in these ATP and mtDNA analyses were from 10–12 month old mice and 12–14 month old hamsters.

### Collection of MII Oocytes Ovulated In Vivo

Female mice and hamsters selected at random with regard to estrous were induced to superovulate by intraperitoneal injections of pregnant mare's serum gonadotropin (PMSG) followed 48 h (mouse) or 54 h (hamster) later by hCG. Dosage for both hormones was 5 IU/mouse or 5 IU/25 g/hamster. Superovulated females were sequentially euthanized over a period of 2 h starting approximately 16 h after the hCG injection. Thus, all oocytes, both young and old mouse and hamster, were collected within 16–17 hours after hCG injection. Each egg mass irrigated from excised oviducts in media (mKrebs for mouse or BWW for hamster) was placed in a 100 µL droplet of medium (mKrebs or BWW) with 0.03% hyaluronidase under paraffin oil and evaluated continuously for the cumulus cells to dissociate from the cumulus mass. Eggs were then picked up with a hand-pulled, glass, micropipette (approximate 100 µm lumen ID) and washed 3 times with hyaluronidase-free medium by vigorous pipeting to remove all remaining cumulus cells. Normal M II stage eggs were evaluated at 32x magnification and collected from the third wash droplet with a micropipette and dispensed into appropriate tubes for either (1) freezing for ATP and mtDNA analysis or (2) transmission electron microscopy (TEM).

### Quantitation of ATP

The ATP content of individual oocytes was determined using the ATP bioluminescent somatic cell assay kit (Sigma, USA). Oocytes were collected, lysed, and stored as above in 100 µl of somatic cell ATP releasing reagent. A volume of 100 µl of 1∶5 diluted ATP assay mix was added to individual wells in an opaque 96-well plate and kept at room temperature for 3–5 minutes to allow endogenous ATP hydrolysis. In a separate tube, 100 µl of ice-cold somatic cell ATP-releasing reagent and 50 µl of ultrapure water were mixed with 50 µl of samples or standards. One half (100 µl) of this mixture was transferred to the reaction wells and the amount of light emitted was immediately measured using a Dynex MLX microtiter plate luminometer. The background luminescence was subtracted from all readings. ATP in single oocyte samples was calculated by comparison to a standard curve generated over the range 2.5–500 fmol/100 µl.

### Preparation of the External Mitochondrial DNA Standard

Quantitative real time PCR using the Roche Lightcycler 480 system was used to quantify mtDNA copy numbers. PCR primers were designed using the mitochondrial 12S ribosomal DNA sequences for golden hamster and C57BL/*6* mice. The hamster 12S rDNA primer sequences are 5′TCAAAGGACTTGGCGG3′ (forward) and 5′CTTCCAGCCCATAGG3′ (reverse), and for mouse, 5′ACCTCACCATCTCTTGC3′ (forward) and 5′GTGTGTGCGTACTTCAT3′ (reverse). All primers were purchased from Integrated DNA Technologies. We first tested the specificity of the selected primers by conventional PCR (1 cycle of denaturation at 95°C for 4 minutes, 35 cycles of amplification at 95°C for 30 sec, 55°C for 30 sec, 72°C for 30 sec and 1 cycle of extension at 72°C for 1 min). The 12S mitochondrial rDNA amplicons (192 bp for hamster and 256 bp for mouse) were subcloned into pCR2, a TOPO-cloning vector (Invitrogen, USA). Recombinants were isolated, purified plasmid DNA was obtained using a Qiagen Plasmid Isolation kit according to the manufacturer’s instructions, and the inserted mtDNA sequence was confirmed by DNA sequence analysis. The purified plasmid DNAs (pFCHam12S and pFCMus12S) were quantified by spectrophotometry at 260 nm.

### Real-Time PCR Quantification of Mitochondrial DNA

The LightCycler DNA FastStart SYBR Green kit (Roche) was utilized to perform real time PCR. Two microliters of oocyte lysate were used as a template in a 20 µl final volume. The DNA was denatured at 95°C for 10 minutes, then amplification proceeded for 45 cycles at 95°C for 0 sec, 55°C for 5 sec and 72°C for 12 sec, followed by melting curve analysis to detect mis-priming at 95°C for 0 sec, 65°C for 15 sec and 95°C for 0 sec for 1 cycle. A standard curve from 10^1^–10^8^ DNA molecules was generated by serial 10-fold dilutions of the appropriate purified stocks of pFCHam12S or pFCMus12S. Relative mtDNA copy numbers for individual oocytes were extrapolated from the standard curve.

### Preparation of Oocytes for TEM

Oocytes were prepared for TEM essentially as described by Britton et al. [43]. Briefly, after overnight fixation in 2.5% glutaraldehyde (GA) at 4°C, oocytes were washed twice in 0.1 M PBS and once in 10% BSA, transferred to a Beem capsule containing 1 drop of 10% BSA and centrifuged. After addition of GA and re-centrifugation, the capsule was filled with GA and refrigerated overnight. After removal of the GA, the pellet was washed twice in 0.1 M PBS and fixed by osmium for 2 hr, then washed twice in PBS. The pellet was then ethanol dehydrated and embedded in EMBed 812. Thin sections were collected on G200-Cu grids and examined under a JEM-1200EXI electron microscope.

### Statistical Analysis

Mean values of ATP content and mitochondrial DNA copy numbers in single oocytes as well as quantitation of mitochondria by transmission electron microscropy were compared using Student t-test between young and old animals. The correlation analysis between ATP content and mitochondrial DNA copy numbers in the same oocytes were calculated using the Pearson correlation test. A p-value <0.05 was considered of biological significance between the groups. Analysis of mitochondrial cristae alterations and vacuolization was analyzed by Student’s t-test after transformed to arcsine.
